# Yeast Strains and Wort Color as Factors Affecting Effects of the Ethanol Fermentation Process

**DOI:** 10.3390/molecules27133971

**Published:** 2022-06-21

**Authors:** Justyna Paszkot, Joanna Kawa-Rygielska

**Affiliations:** Department of Fermentation and Cereals Technology, The Faculty of Biotechnology and Food Science, Wrocław University of Environmental and Life Sciences, 50-630 Wrocław, Poland; joanna.kawa-rygielska@upwr.edu.pl

**Keywords:** fermentation, brewing, yeast, dark beers, dark malts

## Abstract

Dark malts used in the production of brewing wort affect the ethanol fermentation process, the phenolic content, antioxidant capacity and the physiology of yeast cells. An innovative element of this research is the combination of investigating the effect of beer wort color modulated by the use of dark specialty malts on the course and effects of fermentation and the characteristics of post-fermentation yeast biomass of brewer’s strains with different characteristics. Dark and pale beer were obtained. The beers had different ethanol contents (4.51–5.79% *v*/*v*), resulting from real (62.29–80.36%) and apparent (75.37–98.26%) attenuation levels. Metabolic and morphological differences were demonstrated in the brewer’s yeast strains used. *S. cerevisiae* var. *diastaticus* was distinguished by its ability to ferment dextrin, resulting in the highest ethanol content in beers. The total phenolic content in beer depends on the color of the wort and the yeast strain used (244.48–547.56 mg of gallic acid/L). Dark beers show higher ferric ion reduction ability (FRAP) and antioxidant capacity (ABTS^•+^) than pale beers fermented with the same yeast strains. Through biomass analysis, differences in yeast cell physiology depending on yeast strain and beer wort color were also revealed.

## 1. Introduction

Beer is an alcoholic beverage made mainly from water, malt, and hops. The variety of raw materials gives a wide range of possibilities for creating beer recipes. Color is one of the main determinants of beer quality. It depends on the malts used and their degree of drying and roasting, as well as on thermal treatment during wort processing [1]. The Maillard initiated by reaction between reducing sugars and amino acids in the wort during its exposure to elevated temperature is responsible for shaping the intensity of color, as well as other sensory descriptors of beer [2,3]. Pale malts, such as pilsner, pale ale, and wheat, are the base material in the production of beer. Special malts are used in smaller doses to add color and enrich the sensory qualities of beer. The term specialty malts includes malts that, as a result of more intense and longer thermal processing, are characterized by intense caramel, chocolate, or roasted flavor and aroma. The use of specialty malts in the production of brewing wort has a direct effect on the composition of the wort, but also on the metabolism of yeast [1].

Among the strains used in the brewing, we can distinguish two main groups: bottom fermenting yeast (*Saccharomyces pastorianus*) and top fermenting yeast (*Saccharomyces cerevisiae*). Yeasts differ in their metabolic capacities and therefore produce beers with different sensory and physicochemical characteristics. *Saccharomyces cerevisiae* kveik type yeast used in the production of traditional Norwegian beers *and Saccharomyces cerevisiae* var. *diastaticus* used in the production of Belgian saison-style beers are distinct from classic brewer’s yeast strains [4,5]. *S. cerevisiae* kveik yeast shows a high ability to flocculate and rapidly attenuate wort sugars under high temperature conditions (>28 °C). A characteristic feature of *S. cerevisiae* var. *diastaticus* is glucoamylase activity that allows the utilisation of dextrins, which are one of the main carbohydrates in wort. Dextrins are not fermented by the classical strains used in brewing [5,6]. Both kveik yeast and strains used in saison beer production are capable of very efficient utilisation of wort sugars but are incapable of carrying out hydroxycinnamic acid transformations, which are the cause of unfavourable beer aroma characteristics, the so-called phenolic off flavour. These features make yeast interesting for use in the production of beers characterized by a high level of dryness and low calorific value [5].

The purpose of this study was to analyze the influence of the selection of biological material and the use of special dark malts on the effects of the ethanol fermentation process carried out by different yeast strains. Analysis of carbohydrate profiles, content of fermentation products (ethanol and glycerol), pH value, extract and degree of attenuation of worts and beers was performed to characterize the physico-chemical properties of the obtained samples. Wort and beer were subjected to analysis of the content of total phenolic compounds and antioxidant activity. The morphological characteristics of yeast cells in postfermentation biomass was examined using a Scepter Cell Counter cell analyzer. The study provides new information on the characteristics of dark and pale beers produced with different yeast strains, as well as differences in the course and effects of the fermentation process depending on the yeast strain and the color of the beer.

## 2. Results and Discussion

### 2.1. Physicochemical Parameters, Carbohydrates and Glycerol Content

Our research strategy involved the production of a series of dark and pale beers using different strains of brewer’s yeast in the fermentation process. Top-fermenting and bottom-fermenting yeasts were used, among which *Saccharomyces cerevisiae* kveik type and *Saccharomyces cerevisiae* var. *diastaticus* were distinguished for their metabolic capabilities.

**Figure 1:**
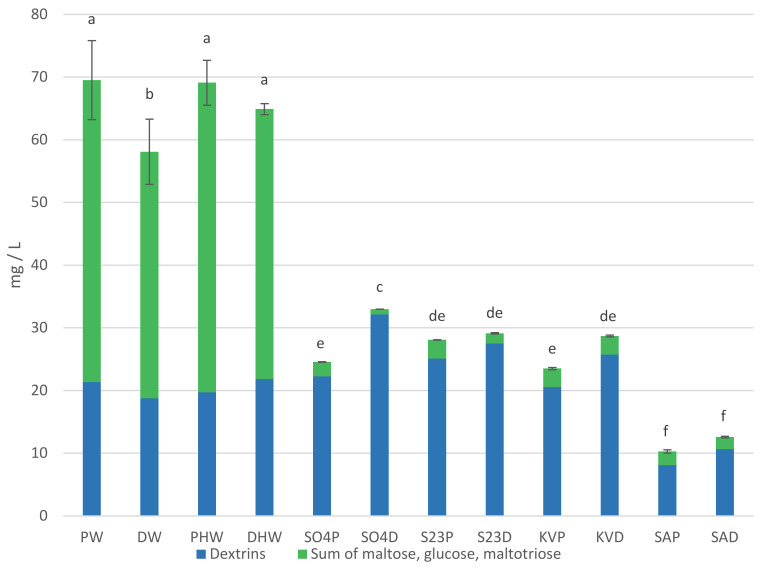
Sum of carbohydrates (glucose, maltose, maltotriose) and dextrins in worts and beers. Values are expressed as mean ± standard deviation. Different letters indicate significant differences between values (n = 2, *p* < 0.05).

Carbohydrates and glycerol content in worts and beers are presented in Table 1 and Figure 1. Maltose, maltotriose, glucose, and dextrins are the main carbohydrates of beer wort. They are mainly obtained by the amylolytic decomposition of starch during malt mashing. The content of individual sugars in the wort depends on the composition and amount of sugars in the malt, the occurrence of non-enzymatic browning reactions and enzyme activity during mashing [1,7]. The proportion of sugars in the wort is influenced by the composition of the mash. The addition of dark chocolate malt at a dose of 10% did not result in significant changes in maltose, maltotriose, and glucose content compared to pale wort [6]. In our investigation, there were no significant differences in maltotriose content between HPW (hopped pale wort) and HDW (hopped dark wort). HPW contained more maltose and glucose than HDW. These sugars are classified as reducing sugars and are involved in Maillard reactions, which may be the reason for their lower content in dark worts [7]. Although DW (dark wort) had a higher extract than PW (pale wort), a lower content of all individual sugars tested was identified in this wort. The sum of the fermentable sugars accounted for 62.93% of the HPW and 56.85% of the HDW extract content. The remaining components of the wort extract are mainly compounds other than fermentable sugars. This is the direct reason for the differences in attenuation rates between pale and dark beers. This is consistent with the observations of other authors [1,6].

The beer variants were not statistically different in terms of maltose, maltotriose, and glucose content. Glucose as a simple sugar is utilized by yeast first. Next, among the sugars tested, the yeast utilizes maltose followed by maltotriose [8]. Dextrins are not fermented by the classical strains of top and bottom fermenting brewing yeasts (S04, S23) and kveik yeast (KV). An exception is the yeast *S. cerevisiae* var. *diastaticus* (SA), which due to the activity of the enzyme glucoamylase has the ability to metabolize dextrins by fermentation [5]. This potential was confirmed in our study. SAP (pale beer fermented with *S. cerevisiae* var. *diastaticus*) and SAD (dark beer fermented with *S. cerevisiae* var. *diastaticus*) beers contained 8.08 mg/L and 10.66 mg/L of dextrins, respectively, while the corresponding HPW and HDW hopped worts contained 19.69 mg/L and 21.84 mg/L. The glucose content in the beers analyzed was 0.15–0.60 g/L. The glucose content of the beers may have resulted from its use for refermentation. The residual amount of sugars in the beers was probably related to the attenuation ability of the yeast strains used.

Glycerol is the main metabolic byproduct of *S. cerevisiae* yeast cells. Its content in beers depends on the course of fermentation and the physiological state of the yeast used in the process. Increased glycerol production is a response to osmotic stress. Therefore, it can be an indicator of the physiological state of yeast during fermentation. Its content in beer can positively influence the viscosity and smoothness sensation of beer during drinking [8]. In our beers, the glycerol content was 1.37–2.05 g/L. The yeast strain that produced beer with the highest glycerol content regardless of beer color was *S. cerevisiae* var. *diastaticus*.

Table 2 shows the results of the analysis of physicochemical parameters of beer. The ethanol content of the beers ranged from 4.51 to 5.79% *v*/*v*. Beers with the highest ethanol content were obtained using the yeast strain *S. cerevisiae* var. *diastaticus.* The influence of beer color on the degree of fermentation was observed. Pale beers were characterized by a higher real degree of fermentation (RDF) as well as apparent degree of fermentation (ADF) regardless of the strain of brewer’s yeast used in the fermentation process. As a result of the high level of attenuation, lower real and apparent extract values were also observed for pale beers. The SAP had the highest attenuation level and the S23D (S23D—dark beer fermented with *S. pastorianus*) the lowest. The beer with the lowest attenuation level was also characterized by the highest real and apparent extract value and the highest caloric value. Beers and S04P (pale beer fermented with *S. cerevisiae*) and KVP (pale beer fermented with *S. cerevisiae* kveik strain) had the lowest caloric value. Dark beers due to lower attenuation and higher wort extract and real extract had higher caloric value. The pH values of the beers ranged from 3.99 to 4.44. The lowest pH values were characteristic for SAD, KVP, and KVD (dark beer fermented with *S. cerevisiae* kveik strain), and the highest were for S23P (pale beer fermented with *S. pastorianus*) and S23D. The dark beer fermented with the KV strain was characterized by a significantly lower pH than the corresponding pale beer. By using different yeast strains, we obtain products with different pH values, which affects the properties of the finished product.

The degree of attenuation of beers is not only the result of the yeast’s potential to utilize the sugars of the wort but is also related to the ability to flocculate. Flocculation is the phenomenon of yeast forming aggregates of cells, resulting in them sedimenting to the bottom of the fermentation vessel. A strong ability to flocculate early in fermentation can result in beers with lower levels of attenuation [9]. The yeast strains we used differ in these capacity, which affects the fermentation process and the characteristics of the resulting beers. *S. cerevisiae* var. *disataticus* has a low flocculation potential, leading to SAP and SAD beers being characterized by the highest RDF and ADF, as well as the highest ethanol content. The other yeast strains, S04, S23, and KV, are characterized by flocculation capacity, which was reflected in the results of RDF, ADF, and the apparent extract content of beers.

### 2.2. Phenolic Compound Content and Antioxidant Activity of Worts and Beers

Table 3 shows the results of the analysis of worts and beers for total phenolic content (TPC) and antioxidant properties (FRAP—ferric reducing antioxidant power, ABTS^+•^—2,2′-azino-bis(3-ethylbenzothiazoline-6-sulfonate) diammonium salt, DPPH^•^—2,2-Diphenyl-1-picrylhydrazyl). Both dark worts and dark beers have higher TPC, FRAP, and ABTS^+•^ than analogous pale worts and beers made with the same yeast strain. The ability to reduce DPPH^•^ radicals is also higher for dark than for pale worts. The antioxidant potential DPPH^•^ for beers was not statistically significantly different depending on the color for the variants S23P and S23D and SAP and SAD. The remaining samples showed a higher value of DPPH^•^ capacity in pale beers than in dark beers.

Dark beers fermented with the same yeast strain had a higher TPC compared to analogous pale beers by 20.62% to 123.96%. Similarly, for FRAP and ABTS^+•^—for each beer analyzed made with the same yeast strain, dark beers showed higher antioxidant activity than pale beers. The FRAP of dark beers was higher than that of pale beers by 60.19–69.07%, ABTS^+•^ from 39.86 to 48.80%. In turn, DPPH^•^ radical scavenging activity did not show significant differences between the dark and pale beer variants S23P and S23D and SAP and SAD, while dark beers made with the top fermentation yeast S04P and the kveik yeast KVD had lower DPPH˙ value than the corresponding pale beers.

Brewing malt and hops are sources of antioxidant compounds in beer: phenols and melanoidins. The amount and type of phenols present in beer affect the sensory properties, shelf life, and colloidal stability of beer during storage. Polyphenolic compounds in a beer occur in a free or bound form [10]. Melanoidins are macromolecular compounds formed in the final phase of the Maillard reaction which are responsible for the resulting color of brewing malt during thermal processes, as well as color changes during the boiling of brewing wort [6]. They affect the antioxidant properties of beers, which explains the higher antioxidant activity of dark beers compared to pale beers.

DW and HDW show higher TPC and FRAP and ABTS^+•^ than pale worts. As the color of the brewing wort darkens, TPC and the antioxidant activity increase [6]. The conditions of the technological process of beer production contribute to a number of changes in the composition of phenolic compounds, which are related to the temperature of the processes, active enzymes of malt, hops, or bioprocesses that occur with microorganisms during fermentation [10,11,12]. As evaluated in our previous studies [11], TPC in beers increases during the hopping and fermentation stages, while it decreases after maturation. According to Fumi et al. [13], fermentation leads to a 16% decrease in TPC due to the adsorption of phenolic compounds on the surface of negatively charged yeast cell walls. This is also supported by reports on the identification of phenolic compounds in waste brewing yeast biomass [14].

Yeast strains used in fermentation differentiate beers in polyphenol content and antioxidant activity [4]. The reason for these variations may be a different cell wall structure. The amount of phenolic compounds adsorbed by yeast increases with the mannan content in the cell wall structure, which depends on the yeast strain. [10]. Mannoproteins, which are one of the main building blocks of the yeast cell wall, located mainly in the outer layer of the cells, play a major role in the determination of the cell charge. During the logarithmic growth phase of yeast cells, a higher mannan content is observed in the cell wall composition compared to the stationary and death phases. As a result of the reduced physiological activity of cells, the mannanoproteines content decreases, so an increase in the content of TPC and individual phenolic compounds in beer is observed towards the end of fermentation [15]. These are released from the mannan-polymer structures. Differences in TPC between beers made with different yeast strains may be due to the physiological state of the cells. The poorer physiological state of biomass after fermentation of dark beers may be due to the content of Maillard reaction compounds that are toxic to yeast. Cells exposed to unfavorable conditions may release some of the adsorbed phenols, resulting in higher phenolic content in dark beers.

### 2.3. Evaluation of the Morphological Characteristics of Yeast Cells

**Figure 2:**
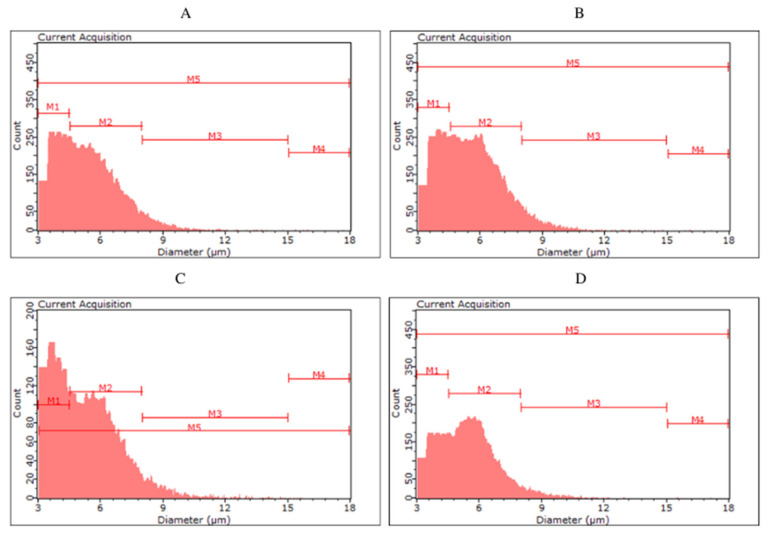
Histograms of yeast cell size in the yeast biomass after the main fermentation by the top fermentation yeast *Saccharomyces cerevisiae* ((**A**)—S04P, (**B**)—S04D) and by the bottom fermentation yeast *Saccharomyces pastoriuanus* ((**C**)—S23P, (**D**)—S23D).

**Figure 3:**
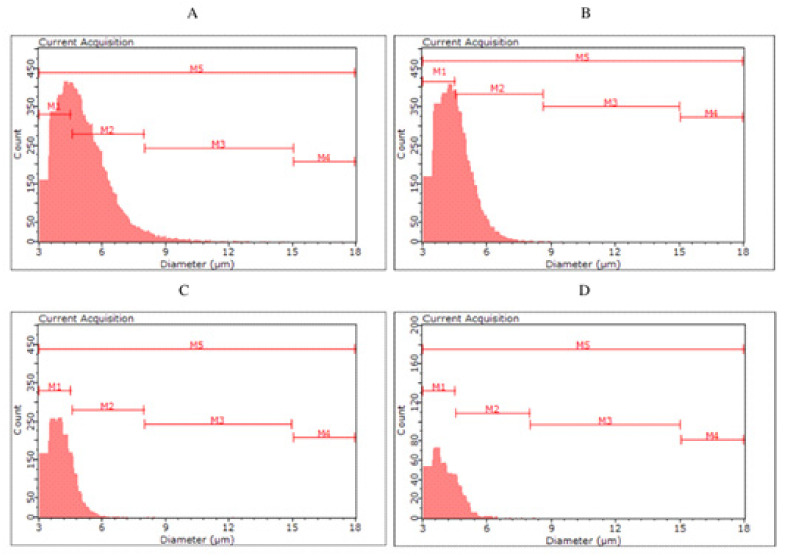
Histograms of yeast cell size in yeast biomass after main fermentation by *Saccharomyces cerevisiae* kveik ((**A**)—KVP, ((**B**)—KVD) and *Saccharomyces cerevisiae* var. *diastaticus* ((**C**)—SAP, (**D**)—SAD).

The yeast cells of the tested strains differed in size and volume (Table 4, Figure 2 and Figure 3). The largest diameter was characteristic for the top fermentation yeast *S. cereviasiae* and the bottom fermentation yeast *S. pastorianus*. No differences in cell volume were observed between lower and upper fermentation yeasts. *The S. cerevisiae* type kveik yeast was characterised by a cell diameter ranging from 4.54 µm for KVD to 5.43 µm for KVC and showed the highest variation in diameter and cell volume depending on the color of fermented beer. The smallest cells among the yeasts were characterised by *S. cerevisiae* var. *diastaticus* strain.

For the biomass collected after fermentation of S04P and S04D (S04D—dark beer fermented with *S. cerevisiae*), no clear differences were observed between the classes. The highest number of cells (56.98–61.19%) were classified in the M2 group, with less in M1 (32.43–37.26%), M3 (5.57–6.25%) and M4 (0.13–0.19%). Among yeast cells in S23P and S23D, we can observe a more pronounced difference in size distribution. In S23P, the number of cells in the M2 was 51.11%, while in S23D it was up to 62.50%. On the other hand, S23P biomass was characterized by a higher proportion of smaller cells—in the M1 class (42.59%) compared to S23D biomass (32.04%). The color of fermented beer had the greatest effect on cell size in the case of KVP and KVD variants, as well as SAP and SAD. The kveik yeast collected after dark beer (KVD) fermentation had a higher proportion of smaller cell size (M1—70.74%) than biomass from KVP beer (M1—43.80%). More cells in the M2 class were observed in the KVP biomass (KVP—52.96%, KVD—28.76%). Most of the *S. cerevisiae* var. *diastaticus* cells were characterized by a size in the range of 4.5–8.0 µm (M1)—84.90% of SAP biomass and 80.96% of SAD biomass cells were classified in the M1 class.

Monitoring the physiological state and morphological characteristics of yeast cells is of importance to the brewing industry. Part of the assessment of the physiological status of yeast cells is measuring their size. The stress conditions for yeast during the fermentation process can promote changes in yeast cell size. Kawa-Rygielska et al. [16] determined that the size and volume distribution of *S. cerevisiae* distillery yeast cells, as well as the concentration of cells in the media after fermentation, depend on the ethanol concentration. As the initial concentration of ethanol in the medium increased, the distribution of cell numbers in size classes changed. They also observed an increase in the number of small cells (M1) and the largest cells (M4) associated with an increase in the ethanol content in the medium. Foszczyńska et al. [17] showed that yeast differed in morphological characteristics depending on the strain used, and ethanol and glucose content in the medium. An increase in ethanol content in the medium favored yeast cells of smaller sizes. Our study confirms that a higher ethanol content causes the appearance of yeast cells with a smaller size. The highest proportion of small cells (M1) was observed in SAP and SAD, which were characterized by the highest ethanol content among all variants analyzed. In the KVD, significantly more cells were recorded in the M1 class than in the KVP. This may be a result of the influence of Maillard reaction compounds on microbial growth and development, caused, among other things, by the chelation of magnesium ions, which are an important factor in yeast metabolic processes [7].

## 3. Materials and Methods

### 3.1. Biological Material

In the ethanol fermentation process of the worts, the following 4 strains of brewer’s yeast were used: *Saccharomyces cerevisiae* “Safale S-04” (Fermentis, Lasaffre, France), *Saccharomyces pastorianus* Saflager S-23 (Fermentis, Lasaffre, France), *Saccharomyces cerevisiae* “Voss” (Lallemand, Canada), *Saccharomyces cerevisiae* var. *diastaticus* “Belle Saison” (Lallemand, Canada).

*Saccharomyces cerevisiae* ‘Safale S-04’ is a yeast strain with high flocculation ability used for English-style beers. The recommended fermentation temperature is 18–26 °C [18].

*Saccharomyces pastorianus* ‘Saflager S-23’ is a yeast used in the production of German style beers. This strain is characterized by a high capacity for flocculation and attenuation. The recommended fermentation temperature is 12–18 °C [19].

*Saccharomyces cerevisiae* ‘Voss’ is one of the kveik yeast strains used in the production of traditional Norwegian beers, a strain selected by Lallemand from a heterogeneous culture of yeast and bacteria that has been cultivated using traditional methods since 1980. *Saccharomyces cerevisiae* ‘Voss’ kveik yeast shows a very high flocculation capacity with a medium to high attenuation level. The recommended fermentation temperature is 18–26 °C [20].

*Saccharomyces cerevisiae* var. *diastaticus* “Belle Saison” is a yeast strain used in the production of Belgian-style beers. It has a low flocculation ability, which allows for beers with a high attenuation level. The recommended fermentation temperature is 35–40 °C [21].

### 3.2. Abbreviations

The following abbreviations were used to mark the samples: PW—pale wort, DW -dark wort, HPW—hopped pale wort, HDW—hopped dark wort, S04P—pale beer fermented with *S. cerevisiae*, S04D—dark beer fermented with *S. cerevisiae*, S23P—pale beer fermented with *S. pastorianus*, S23D—dark beer fermented with *S. pastorianus*, KVP—pale beer fermented with *S. cerevisiae* kveik strain, KVD—dark beer fermented with *S. cerevisiae* kveik strain, SAP—pale beer fermented with *S. cerevisiae* var. *diastaticus*, SAD—dark beer fermented with *S. cerevisiae* var. *diastaticus*.

### 3.3. Brewing Technology

The technological process for the production of dark and pale beers was carried out considering the following technological stages: infusion mashing, mash filtration, batch sparge with water (72 °C), hopping (60 min, 100 °C), cooling (20 °C), filtration, division into 2 L samples, inoculation and fermentation. The malt mash was composed of pale beer from 100% (3.3 kg) pilsner barley malt (Viking Malt, Strzegom, Poland) and for dark beer from 90% (3.1 kg) pilsner barley malt and 10% (0.34 kg) chocolate dark barley malt (Viking Malt, Strzegom, Poland).

For mashing, 3.5 L of water per kilogram of malt was used. Mashing was carried out as follows: 52 °C for 10 min, 63 °C for 40 min, 72 °C for 30 min and 78 °C for 10 min [11]. The temperature rise was achieved at a rate of 1 °C/min. Marynka bitter hops in a dose of 17 g for 60 min of boiling (Marxam, Krakow, Poland) and 17 g aroma hops Lubelski for 10 min (Marxam, Krakow, Poland) were used for wort hopping. After cooling, pale hopped wort (HWP, 8.39 ± 0.36 EBC, extract 10.78 ± 0.13 °Bx) and dark hopped wort (HWD, 85.71 ± 1.67 EBC, extract 11.45 ± 0.18 °Bx) were obtained.

The dried yeast biomass was rehydrated prior to wort inoculation. Worts with a volume of 2 L were inoculated with one of 4 selected strains of brewing yeast (1 g of dried biomass per 1 L of wort). Fermentation was carried out in a laboratory incubator for 7 days at the following temperatures: 18 °C for S04P and S04D, 12 °C for S23P and S23D, 35 °C for KVP and KVD and 18 °C for SAD and SAP. The beers were then decanted from above the sediment and placed in sterile fermentation tanks. Post-fermentation was carried out for 7 days. The beers were bottled in 0.5 L bottles with glucose (2 g/L) to allow refermentation. The ageing of the beer was carried out at 4 °C for 28 days.

### 3.4. Basic Physico-Chemical Parameters

Analysis of ethanol content, real attenuation degree (RDF, % *w*/*w*) and apparent attenuation degree (ADF, % *w*/*w*), wort extract content (% *w*/*w*) and apparent beer extract (% *w*/*w*), color (EBC), density (g/cm^3^) and caloricity (kcal/100 mL) were performed using an oscillating densitometer with a beer analyzer DMA 4500 M (Anton Paar, Graz, Austria) [4]. The beers were shaken for 20 min before analysis using a 358 A laboratory shaker (Elpin Plus, Lubawa, Poland). Diatomaceous earth was added at a dose of 1 g/100 mL to the degassed beers and shaking was continued for 10 min. The samples were filtered using paper strainers. The results presented are the mean of two replications of the analysis. The pH of the beers was determined in triplicate using a Mettler Toledo MP 240 pH meter (Columbus, OH, USA). The analyzer determined by the analyzer based on density (ρ), reak extract (Er) and alcohol content (A) according to the formula: Caloricity [kcal/100 mL] = 7 ·A [*w*/*w*] + 3.5 Er [*w*/*w*]·ρ [g/cm^3^].

### 3.5. High-Performance Liquid Chromatography Analysis of Carbohydrates and Glycerol Content

Maltose, maltotriose, glucose, ethanol, and glycerol were analyzed by high performance liquid chromatography (HPLC) using a Prominence apparatus (Shimadzu, Kyoto, Japan) [4]. Worts and beers were degassed by shaking using a 358 A laboratory shaker (Elpin Plus, Lubawa, Poland) and centrifuged (10 min, 5000 rpm, MPW-351R centrifuge). A three-fold dilution of worts and a two-fold dilution of beers were used. The diluted samples were filtered through 0.22 µm syringe filters. Separation was carried out using a Rezex ROA-Organic Acid H+ column (300 × 7.8 mm^2^) (Phenomenex, Torrance, CA, USA), a temperature of 60 °C, a flow rate of 0.6 mL/min and an injection volume 0.02 mL. The mobile phase was a solution of 0.005 mol/dm^3^ H_2_SO_4_. The isocratic elution and the refractometric detection method were used. The analysis was performed in duplicate. The five-point calibration curve integrated in Chromax 10.0 software (Pol-Lab, Wilkowice, Poland) was used to determine the analyte concentration. The results were presented as g/L of wort or beer.

### 3.6. Total Polyphenols Content

The Folin-Ciocalteu (F-C) spectrophotometric method was used to determine total polyphenols (TPC) in worts and beers [22]. In plastic cuvettes, 0.1 mL of sample and 0.2 mL of F-C reagent were mixed. After 3 min, 1 mL of 20% aqueous sodium carbonate solution and 2 mL of distilled water were added. The samples were incubated for 1 h. The absorbance was analyzed spectrophotometrically using a UV-2401 PC (Shimadzu, Kyoto, Japan) at 765 nm. Distilled water was used as a blank. Results are presented as the average value of three replicates. A calibration curve in the range of 0.30–9.00 mg GAE/L was used to read the results. The results were expressed as gallic acid equivalents (GAE) per liter of beer or wort.

### 3.7. Ability of Iron Ion Reduction (FRAP)

The analysis of ferric ion reducing ability was performed [23]. The ferric reducing antioxidant power (FRAP) reagent was a mixture of 20 mL of aqueous ferric (III) chloride solution (0.1018 g FeCl_3_) with a solution of 2,4,6-tris (2-pyridyl)-s-triazine (0.0664 g TPTZ) in 40 mM hydrochloric acid (20 mL HCl) with acetate buffer (pH 3.6). Samples diluted in distilled water (1 mL) and 3 mL of FRAP reagent were placed in cuvettes. The absorbance was read at 593 nm using a UV-2401 PC spectrophotometer (Shimadzu, Kyoto, Japan). Distilled water was used as a blank. The results were determined from a calibration curve in the range of 1.25–12.50 µmol TE/L. Results were presented as the average of three replicates in millimoles of Trolox (TE) per liter of wort or beer.

### 3.8. Ability of Radical Cation ABTS^•+^ Reduction

Antioxidant activity was determined using the ABTS^•+^ (2,2′-azino-bis(3-ethylbenzothiazoline-6-sulfonic acid) cation radical reduction method [24]. Wort or beer (0.03 mL) were mixed in cuvettes with a solution of ABTS^•+^ with a measured absorbance value (0.700). The incubation was carried out for 6 min. The absorbance was measured using a UV-2401 PC spectrophotometer (Shimadzu, Kyoto, Japan) at 734 nm. Determinations were made in triplicate. The results were determined from a calibration curve drawn in the range of 1.70–21.70 µmol TE/L and presented in mmol Trolox (TE) per liter of wort or beer.

### 3.9. Ability of Radical Cation DPPH^•^ Reduction

Antioxidant capacity analysis was performed using the DPPH^•^ (2,2-diphenyl-1-picrylhydrazyl) method [25]. In cuvettes, 0.1 mL of wort or beer was mixed with 2 mL of a 0.04 mmol/L DPPH^•^ solution in methanol and 0.4 mL of H_2_O. The samples were incubated for 10 min. Absorbance was measured with a spectrophotometer at 517 nm. During the DPPH^•^ radical reduction reaction, the color of the solution changed from purple to yellow. A calibration curve was prepared using Trolox solution (2–10 µmol TE/L). The results were presented in millimoles of Trolox (TE) per liter of wort or beer. All measurements were made in triplicate.

### 3.10. Evaluation of the Morphological Characteristics of Yeast Cells

Analysis of the morphological characteristics and number of yeast cells after the fermentation process was performed using a Scepter Cell Counter cell analyzer equipped with 40 µm sensors (Merck, Darmstadt, Germany). The biomass of the brewery yeast was collected after the main fermentation process, then centrifuged 3 times (each time separating the supernatant and resuspending the cells in saline). A buffered saline solution was then prepared. To 100 mL of 0.9% NaCl was added 1 tablet of tris buffered saline (TBS) (Merck, Darmstadt, Germany). The diluted biomass (100 µL) was collected in a 1.5 mL Eppendorf tube, then 1 mL of TBS was added and centrifuged using a laboratory micro centrifuge for 10 min. Centrifugation was repeated 3 times; each time, the supernatant was separated and cells were resuspended in 1 mL of TBS. The sample was analyzed. The results were presented as cell size divided into classes according to the method described by Kawa-Rygieska et al. [16]. Cells were classified according to the following criteria: Class I (M1) cells with a diameter of 3.0–4.5 μm, Class II (M2) cells with a diameter of 4.6–8.0 μm, Class III (M3) cells with a diameter of 8.1–15.0 μm, Class IV (M4) cells with a diameter of 15.1–18.0 μm.

### 3.11. Statistical Analysis

A one-way analysis of variability (ANOVA) was performed for the data obtained. The significance of the differences between the mean values was tested using Duncan’s test (*p* < 0.05). Statistical analysis was performed using the Statistica 13.5 program (StatSoft, Tulsa, OK, USA).

## 4. Conclusions

The application of dark specialty malts in production technology plays a key role in shaping the characteristics of beer quality. The addition of dark malts in the production of brewing wort influenced the reduction in the total amount of carbohydrates, while the selection of microorganisms in the fermentation process influenced the composition of sugars in beer. The main carbohydrate identified in the worts and beers was dextrins, regardless of the yeast strain used for fermentation. The yeast *S. cerevisiae* var. *diastacicus* was distinguished from other yeast strains by its ability to attenuate dextrins, resulting in beers with exceptionally high ethanol content. However, the color of the beers affected the level of attenuation, which resulted in the content differences in ethanol and apparent extract, as the well as caloric content between variants. Dark beers were characterized by a lower level of attenuation regardless of the yeast strain used compared to pale beers. As we know from the characteristics of the brewing yeast strains used, they had different flocculation abilities, which affected the level of attenuation and the final quality of the beers. The dark beers studied had a higher total phenolic compound content and antioxidant potential than the pale beers. Furthermore, the beers differed in antioxidant potential depending on the yeast strain used. The dark color of the beers associated with the addition of special malts affected the distribution of cell size in the post-fermentation biomass of bottom-fermenting yeast and kveik yeast. The use of dark wort increased the number of large cells in the biomass of *S. cerevisiae* kveik yeast and decreased their number in the biomass of bottom-fermenting *S. pastorianus* yeast. Investigating the effect of the physiological state during fermentation on antioxidant activity and phenolic compound content when exposed to Maillard reaction compounds and ethanol stress conditions during fermentation is an interesting area for further research.

## Figures and Tables

**Table 1:** Carbohydrate profile and glycerol content of worts and beers.

Compound	Unit	PW	DW	HPW	HDW	S04P	S04D	S23P	S23D	KVP	KVD	SAP	SAD
Maltose	g/L	32.24 ± 2.53 a ^1^	26.47 ± 1.36 c	33.10 ± 1.45 a	30.17 ± 0.40 b	0.36 ± 0.01 d	nd ^2^	0.86 ± 0.00 d	0.66 ± 0.00 d	1.00 ± 0.01 d	0.99 ± 0.02 d	0.97 ± 0.06 d	0.86 ± 0.05 d
Maltotriose		9.61 ± 0.87 a	8.70 ± 2.48 a	9.78 ± 0.66 a	8.17 ± 0.10 a	1.71 ± 0.00 b	0.85 ± 0.00 b	1.84 ± 0.00 b	0.97 ± 0.00 b	1.37 ± 0.01 b	1.51 ± 0.04 b	1.00 ± 0.06 b	0.91 ± 0.04 b
Glucose		6.34 ± 0.53 a	4.16 ± 0.21 b	6.54 ± 0.97 a	4.73 ± 0.05 b	0.23 ± 0.00 c	nd	0.32 ± 0.00 c	nd	0.60 ± 0.01 c	0.48 ± 0.10 c	0.23 ± 0.01 c	0.15 ± 0.00 c
Dextrins		21.34 ± 2.37 de	18.77 ± 1.15 f	19.69 ± 0.51 ef	21.84 ± 0.33 d	22.25 ± 0.05 d	32.13 ± 0.01 a	25.07 ± 0.04 c	27.50 ± 0.10 b	20.55 ± 0.16 def	25.73 ± 0.19 c	8.08 ± 0.15 h	10.66 ± 0.04 g
Glicerol		nd	nd	nd	nd	1.54 ± 0.00 cd	1.92 ± 0.00 ab	2.05 ± 0.00 a	1.35 ± 0.01 d	1.37 ± 0.01 d	1.51 ± 0.15 cd	1.82 ± 0.19 abc	1.68 ± 0.33 bcd

^1^ Values are expressed as mean ± standard deviation. Different letters (a, b, c, etc.) indicate significant differences between values (n = 2, *p* < 0.05), ^2^ nd, not detected. Abbreviations: PW—pale wort, DW—dark wort, HPW—hopped pale wort, HDW—hopped dark wort, S04P—pale beer fermented with *S. cerevisiae*, S04D—dark beer fermented with *S. cerevisiae*, S23P—pale beer fermented with *S. pastorianus*, S23D—dark beer fermented with *S. pastorianus*, KVP—pale beer fermented with *S. cerevisiae* kveik strain, KVD—dark beer fermented with *S. cerevisiae* kveik strain, SAP—pale beer fermented with *S. cerevisiae* var. *diastaticus*, SAD—dark beer fermented with *S. cerevisiae* var. *diastaticus*.

**Table 2:** Physicochemical parameters of beers.

Parameter	Unit	S04P	S04D	S23P	S23D	KVP	KVD	SAP	SAD
Ethanol	% *v*/*v*	4.51 ± 0.02 h ^1^	4.71 ± 0.01 e	4.73 ± 0.01 d	4.66 ± 0.00 f	4.80 ± 0.01 c	4.58 ± 0.01 g	5.60 ± 0.01 b	5.79 ± 0.00 a
Density	g/cm^3^	1.006 ± 0.00 d	1.009 ± 0.00 a	1.006 ± 0.00 e	1.009 ± 0.00 b	1.004 ± 0.00 f	1.008 ± 0.00 c	0.999 ± 0.00 h	1.002 ± 0.00 g
Real extract	% *w*/*w*	3.75 ± 0.00 d	4.38 ± 0.00 b	3.70 ± 0.00 e	4.56 ± 0.00 a	3.34 ± 0.01 f	4.20 ± 0.00 c	2.23 ± 0.00 h	2.58 ± 0.00 g
Apparent extract	% *w*/*w*	2.11 ± 0.01 d	2.68 ± 0.01 b	1.98 ± 0.01 e	2.87 ± 0.01 a	1.58 ± 0.01 e	2.54 ± 0.01 c	0.19 ± 0.01 g	0.47 ± 0.01 f
RDF	% *w*/*w*	66.06 ± 0.11 e	63.44 ± 0.02 g	67.45 ± 0.06 d	62.29 ± 0.01 h	70.03 ± 0.01 c	63.78 ± 0.06 f	80.36 ± 0.04 a	78.56 ± 0.01 b
ADF	% *w*/*w*	80.25 ± 0.13 e	76.81 ± 0.03 g	81.94 ± 0.08 d	75.37 ± 0.01 h	85.23 ± 0.01 c	77.31 ± 0.08 f	98.26 ± 0.04 a	95.89 ± 0.02 b
Calories	kcal/100 mL	38.07 ± 0.12 g	41.47 ± 0.02 b	39.46 ± 0.35 e	41.83 ± 0.01 a	38.24 ± 0.05 g	40.10 ± 0.07 d	38.71 ± 0.06 f	41.01 ± 0.04 c
pH	-	4.21 ± 0.04 b	4.12 ± 0.00 b	4.44 ± 0.01 a	4.42 ± 0.00 a	4.13 ± 0.01 b	4.00 ± 0.01 c	4.04 ± 0.01 c	3.99 ± 0.01 c

^1^ Values are expressed as mean ± standard deviation. Different letters (a, b, c, etc.) indicate significant differences between values (n = 2, *p* < 0.05). Abbreviations: RDF—real degree of fermentation, ADF—apparent degree of fermentation.

**Table 3:** Total phenols content (TPC) and antioxidant activity (FRAP, ABTS^+•^, DPPH^•^) of worts and beers.

Parameter	Unit	PW	DW	HPW	HDW	S04P	S04D	S23P	S23D	KVP	KVD	SAP	SAD
TPC	mg GAE/L	242.54 ± 2.55 h ^1^	316.99 ± 3.87 e	278.65 ± 2.10 f	402.55 ± 7.50 b	286.99 ± 5.85 f	346.16 ± 1.04 d	260.32 ± 3.16 g	365.88 ± 5.87 c	233.09 ± 7.12 i	333.66 ± 3.07 d	244.48 ± 3.37 h	547.56 ± 2.07 a
FRAP	mmol TE/L	0.68 ± 0.02 g	1.28 ± 0.02 c	1.13 ± 0.04 d	1.70 ± 0.02 a	0.97 ± 0.05 f	1.64 ± 0.04 ab	1.03 ± 0.01 e	1.65 ± 0.01 ab	0.99 ± 0.02 ef	1.62 ± 0.01 b	1.01 ± 0.01 ef	1.64 ± 0.01 ab
ABTS^+•^		1.19 ± 0.02 h	1.73 ± 0.01 d	1.31 ± 0.02 f	2.22 ± 0.01 a	1.25 ± 0.02 g	1.86 ± 0.03 c	1.38 ± 0.01 e	1.93 ± 0.04 b	1.21 ± 0.01 g	1.74 ± 0.02 d	1.25 ± 0.04 g	1.76 ± 0.01 d
DPPH^•^		0.31 ± 0.01 d	0.58 ± 0.02 b	0.20 ± 0.04 gh	0.87 ± 0.01 a	0.18 ± 0.02 h	0.04 ± 0.02 i	0.27 ± 0.01 ef	0.30 ± 0.02 de	0.23 ± 0.02 fg	0.01 ± 0.01 i	0.50 ± 0.01 c	0.47 ± 0.01 c

^1^ Values are expressed as mean ± standard deviation. Different letters (a, b, c, etc.) indicate significant differences between values (n = 2, *p* < 0.05).

**Table 4:** Characteristics of the brewing yeast cell biomass using Scepter Cell Counter.

Parameter		Unit	S04P	S04D	S23P	S23D	KVP	KVD	SAP	SAD
Mean cell diameter		µm	5.88	6	5.86	5.89	5.43	4.54	4.15	4.13
Mean cell volume		pL	0.11	0.11	0.11	0.11	0.08	0.05	0.04	0.04
Cell size class	M1	%	37.26	32.43	42.59	32.04	43.80	70.74	84.90	80.96
	M2		56.98	61.19	51.11	62.50	52.96	28.76	14.51	18.54
	M3		5.57	6.25	6.13	5.30	3.16	0.50	0.59	0.51
	M4		0.19	0.13	0.18	0.16	0.08	0.00	0.00	0.00

## Data Availability

The data presented in this study are available on request from the corresponding author.

## Sample Availability

Not available.
